# *tert*-Butyl 3-[2,2-bis­(ethoxy­carbon­yl)vin­yl]-2-bromo­methyl-1*H*-indole-1-carboxyl­ate

**DOI:** 10.1107/S1600536809041567

**Published:** 2009-10-17

**Authors:** M. Thenmozhi, T. Kavitha, V. Dhayalan, A. K. Mohanakrishnan, M. N. Ponnuswamy

**Affiliations:** a Centre of Advanced Study in Crystallography and Biophysics, University of Madras, Guindy Campus, Chennai 600 025, India; b Department of Organic Chemistry, University of Madras, Guindy Campus, Chennai 600 025, India

## Abstract

In the title compound, C_22_H_26_BrNO_6_, the indole ring system is planar [maximum deviation 0.029 (2) Å]. The *tert*-butyl bound carboxyl­ate group forms a dihedral angle of 17.54 (8)° with the indole ring system. In the crystal, mol­ecules are linked into centrosymmetric *R*_2_^2^(10) dimers by paired C—H⋯O hydrogen bonds.

## Related literature

For general background to indoles, see: Gribble (1996 ▶); Jing-Ru *et al.* (2007 ▶); Ximenes *et al.* (2005 ▶). For hybridization, see: Beddoes *et al.* (1986 ▶). For hydrogen-bond motifs, see: Bernstein *et al.* (1995 ▶).
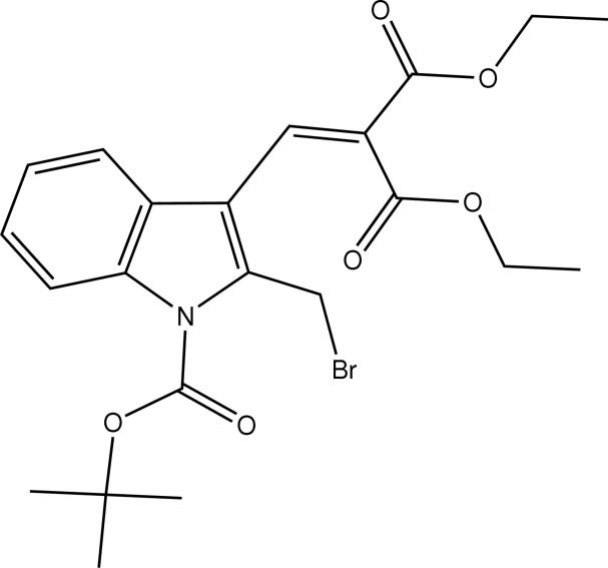


## Experimental

### 

#### Crystal data

C_22_H_26_BrNO_6_
*M* *_r_* = 480.35
Triclinic, 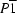

*a* = 10.8682 (3) Å
*b* = 11.1094 (4) Å
*c* = 11.5699 (6) Å
α = 111.984 (3)°
β = 105.841 (2)°
γ = 106.926 (2)°
*V* = 1118.51 (9) Å^3^
*Z* = 2
Mo *K*α radiation
μ = 1.88 mm^−1^
*T* = 293 K
0.30 × 0.25 × 0.20 mm

#### Data collection

Bruker Kappa APEXII area-detector diffractometer
Absorption correction: multi-scan (*SADABS*, Sheldrick, 2001 ▶) *T*_min_ = 0.603, *T*_max_ = 0.706
32165 measured reflections
8669 independent reflections
5490 reflections with *I* > 2σ(*I*)
*R*_int_ = 0.028

#### Refinement

*R*[*F* ^2^ > 2σ(*F* ^2^)] = 0.040
*wR*(*F* ^2^) = 0.114
*S* = 1.01
8669 reflections
271 parameters
1 restraint
H-atom parameters constrained
Δρ_max_ = 0.29 e Å^−3^
Δρ_min_ = −0.60 e Å^−3^

### 

Data collection: *APEX2* (Bruker, 2004 ▶); cell refinement: *SAINT* (Bruker, 2004 ▶); data reduction: *SAINT*; program(s) used to solve structure: *SHELXS97* (Sheldrick, 2008 ▶); program(s) used to refine structure: *SHELXL97* (Sheldrick, 2008 ▶); molecular graphics: *ORTEP-3* (Farrugia, 1997 ▶); software used to prepare material for publication: *SHELXL97* and *PLATON* (Spek, 2009 ▶).

## Supplementary Material

Crystal structure: contains datablocks global, I. DOI: 10.1107/S1600536809041567/ci2911sup1.cif

Structure factors: contains datablocks I. DOI: 10.1107/S1600536809041567/ci2911Isup2.hkl

Additional supplementary materials:  crystallographic information; 3D view; checkCIF report

## Figures and Tables

**Table 1:** Hydrogen-bond geometry (Å, °)

*D*—H⋯*A*	*D*—H	H⋯*A*	*D*⋯*A*	*D*—H⋯*A*
C18—H18*A*⋯O4^i^	0.97	2.56	3.392 (3)	144

Symmetry code: (i) 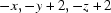.

**Fig. 1.**
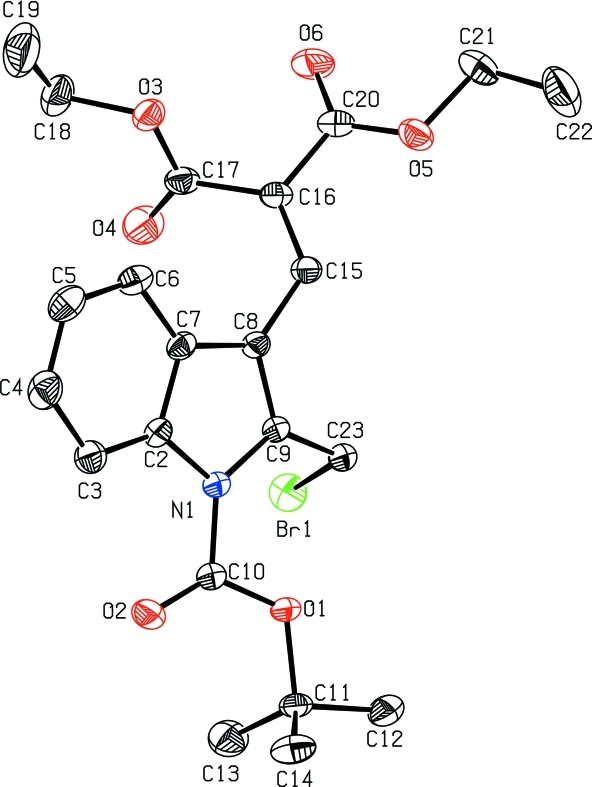
The molecular structure of the title compound. Displacement ellipsoids are drawn at the 30% probability level. H atoms have been omitted for clarity.

**Fig. 2.**
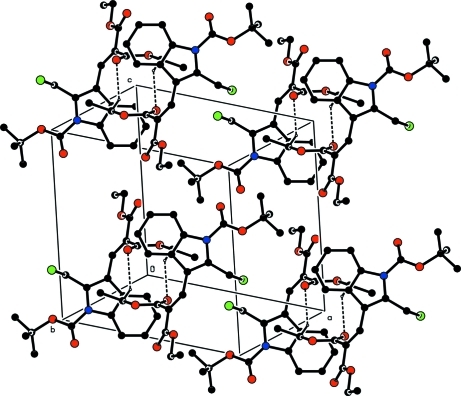
A view of the crystal packing of molecules, showing C–H···O interactions (dashed lines), leading to dimer formation.

## supplementary crystallographic information

### Comment

Indole is a common motif for a drug target and, as such, the development of new diversity-tolerant routes to this previleged biological scaffold continues to be of significant benefit (Gribble *et al.*, 1996) and forms the basis of a wide variety of drugs, including the anti-inflammatory agent indomethacin, reserpine (exploited as hypotensive agent) and sumatriptan (used for the treatment of magraine). The indole derivatives are the effective inhibitors of myeloperoxidase(MPO)-chlorinating activity (Ximenes *et al.*, 2005). Indole-3-carbinol has emerged as a promising chemopreventive agent due to its *in vivo* efficacy in prostate cancer cells of various animal models (Jing-Ru *et al.*, 2007).

The indole ring system of the title molecule (Fig.1) is planar and the bromomethyl group is oriented at an angle of 74.98 (8)°. The *tert* butyl carboxylate group substituted at N1 of the indole ring is in an extended conformation [N1–C10–O1–C11 = 176.24 (16)°]. Both ethoxycarbonyl groups adopt extended conformations as can be seen from torsion angles C16–C17–O3–C18 [-179.61 (16)°], C17–O3–C18–C19 [-156.5 (2)°], C16–C20–O5–C21 [179.18 (14)°] and C20–O5–C21–C22 [-177.9 (2)°]. The sum of bond angles around N1 [360.0 (4)°] indicates that atom N1 exhibits *sp*^2^ hybridization (Beddoes *et al.*, 1986).

The crystal structure is stabilized by C–H···O hydrogen bonds. The molecules form centrosymmetric *R*_2_^2^(10) dimers through paired C18–H18A···O4 hydrogen bonds (Fig. 2) (Bernstein *et al.*, 1995).

### Experimental

A solution of *tert*-butyl 3-(2,2-di(ethoxycarbonyl)vinyl)-2-methyl-1*H*-indole- 1-carboxylate (2 g, 4.98 mmol) in dry carbon tetrachloride (80 ml), azobis(isobutyronitrile)(AIBN) (0.07 g) and finely powdered *N*-bromosuccinimide(NBS) (0.93 g, 5.23 mmol) were added and refluxed for 2 h. Then, the reaction mixture was cooled to room temperature. The floated succinimide was filtered off and washed with carbon tetrachloride (10 ml). The combined filtrate was concentrated *in vacuo* to afford the title compound (1.91 g, 80%) as colourless crystals.

### Refinement

H atoms were positioned geometrically (C-H = 0.93–0.97 Å) and allowed to ride on their parent atoms, with *U*_iso_(H) = 1.5U_eq_(C) for methyl H and 1.2U_eq_(C) for other H atoms. The C18–C19 bond distance was restrained to 1.50 (5) Å.

### Crystal data

**Table:**

C_22_H_26_BrNO_6_	*Z* = 2
*M**_r_* = 480.35	*F*(000) = 496
Triclinic, *P*1	*D*_x_ = 1.426 Mg m^−^^3^
Hall symbol: -P 1	Mo *K*α radiation, λ = 0.71073 Å
*a* = 10.8682 (3) Å	Cell parameters from 8669 reflections
*b* = 11.1094 (4) Å	θ = 2.1–33.8°
*c* = 11.5699 (6) Å	µ = 1.88 mm^−^^1^
α = 111.984 (3)°	*T* = 293 K
β = 105.841 (2)°	Block, colourless
γ = 106.926 (2)°	0.30 × 0.25 × 0.20 mm
*V* = 1118.51 (9) Å^3^	

### Data collection

**Table:**

Bruker Kappa APEXII area-detector diffractometer	8669 independent reflections
Radiation source: fine-focus sealed tube	5490 reflections with *I* > 2σ(*I*)
graphite	*R*_int_ = 0.028
ω and φ scans	θ_max_ = 33.8°, θ_min_ = 2.1°
Absorption correction: multi-scan (*SADABS*, Sheldrick, 2001)	*h* = −17→16
*T*_min_ = 0.603, *T*_max_ = 0.706	*k* = −16→17
32165 measured reflections	*l* = −17→17

### Refinement

**Table:**

Refinement on *F*^2^	Primary atom site location: structure-invariant direct methods
Least-squares matrix: full	Secondary atom site location: difference Fourier map
*R*[*F*^2^ > 2σ(*F*^2^)] = 0.040	Hydrogen site location: inferred from neighbouring sites
*wR*(*F*^2^) = 0.114	H-atom parameters constrained
*S* = 1.01	*w* = 1/[σ^2^(*F*_o_^2^) + (0.0566*P*)^2^ + 0.1525*P*] where *P* = (*F*_o_^2^ + 2*F*_c_^2^)/3
8669 reflections	(Δ/σ)_max_ = 0.001
271 parameters	Δρ_max_ = 0.29 e Å^−^^3^
1 restraint	Δρ_min_ = −0.60 e Å^−^^3^

### Special details

**Table:**

Geometry. All e.s.d.'s (except the e.s.d. in the dihedral angle between two l.s. planes) are estimated using the full covariance matrix. The cell e.s.d.'s are taken into account individually in the estimation of e.s.d.'s in distances, angles and torsion angles; correlations between e.s.d.'s in cell parameters are only used when they are defined by crystal symmetry. An approximate (isotropic) treatment of cell e.s.d.'s is used for estimating e.s.d.'s involving l.s. planes.
Refinement. Refinement of *F*^2^ against ALL reflections. The weighted *R*-factor *wR* and goodness of fit *S* are based on *F*^2^, conventional *R*-factors *R* are based on *F*, with *F* set to zero for negative *F*^2^. The threshold expression of *F*^2^ > σ(*F*^2^) is used only for calculating *R*-factors(gt) *etc*. and is not relevant to the choice of reflections for refinement. *R*-factors based on *F*^2^ are statistically about twice as large as those based on *F*, and *R*- factors based on ALL data will be even larger.

### Fractional atomic coordinates and isotropic or equivalent isotropic displacement parameters (Å^2^)

**Table:**

	*x*	*y*	*z*	*U*_iso_*/*U*_eq_	
C2	0.09184 (16)	0.74325 (17)	1.16827 (16)	0.0342 (3)	
C3	0.07086 (19)	0.8044 (2)	1.28578 (19)	0.0441 (4)	
H3	0.1358	0.8307	1.3725	0.053*	
C4	−0.0521 (2)	0.8242 (2)	1.2671 (2)	0.0537 (5)	
H4	−0.0694	0.8659	1.3435	0.064*	
C5	−0.14938 (19)	0.7836 (2)	1.1379 (2)	0.0528 (5)	
H5	−0.2299	0.7997	1.1293	0.063*	
C6	−0.12946 (17)	0.7201 (2)	1.0217 (2)	0.0427 (4)	
H6	−0.1966	0.6909	0.9349	0.051*	
C7	−0.00584 (15)	0.70013 (16)	1.03705 (17)	0.0341 (3)	
C8	0.04611 (15)	0.63561 (16)	0.94006 (16)	0.0328 (3)	
C9	0.17083 (15)	0.63964 (16)	1.01285 (15)	0.0323 (3)	
C10	0.31912 (16)	0.73467 (18)	1.26701 (16)	0.0365 (3)	
C11	0.51726 (16)	0.67167 (19)	1.32235 (17)	0.0391 (3)	
C12	0.5566 (2)	0.5718 (3)	1.2261 (2)	0.0578 (5)	
H12A	0.4829	0.4747	1.1791	0.087*	
H12B	0.6448	0.5760	1.2782	0.087*	
H12C	0.5671	0.6010	1.1598	0.087*	
C13	0.4815 (2)	0.6177 (3)	1.4162 (2)	0.0565 (5)	
H13A	0.4495	0.6784	1.4716	0.085*	
H13B	0.5647	0.6199	1.4752	0.085*	
H13C	0.4074	0.5202	1.3616	0.085*	
C14	0.6292 (2)	0.8251 (2)	1.3980 (2)	0.0603 (5)	
H14A	0.6402	0.8560	1.3328	0.090*	
H14B	0.7180	0.8316	1.4517	0.090*	
H14C	0.6009	0.8861	1.4582	0.090*	
C15	−0.02203 (15)	0.56833 (17)	0.78999 (16)	0.0361 (3)	
H15	−0.0246	0.4788	0.7407	0.043*	
C16	−0.08096 (15)	0.62017 (18)	0.71535 (16)	0.0367 (3)	
C17	−0.06921 (18)	0.76980 (19)	0.77787 (18)	0.0425 (4)	
C18	−0.1952 (2)	0.9111 (2)	0.8069 (3)	0.0658 (6)	
H18A	−0.1147	0.9770	0.8966	0.079*	
H18B	−0.1864	0.9505	0.7460	0.079*	
C19	−0.3273 (3)	0.8959 (3)	0.8200 (4)	0.1028 (11)	
H19A	−0.4062	0.8172	0.7355	0.154*	
H19B	−0.3367	0.9837	0.8387	0.154*	
H19C	−0.3262	0.8766	0.8944	0.154*	
C20	−0.14814 (17)	0.5379 (2)	0.56162 (18)	0.0428 (4)	
C21	−0.2390 (2)	0.3118 (2)	0.36364 (19)	0.0571 (5)	
H21A	−0.1787	0.3480	0.3245	0.069*	
H21B	−0.3299	0.3122	0.3247	0.069*	
C22	−0.2597 (4)	0.1643 (3)	0.3329 (3)	0.0913 (9)	
H22A	−0.1688	0.1647	0.3688	0.137*	
H22B	−0.3073	0.1009	0.2347	0.137*	
H22C	−0.3165	0.1311	0.3750	0.137*	
C23	0.26606 (17)	0.59735 (18)	0.95525 (16)	0.0373 (3)	
H23A	0.2140	0.5362	0.8558	0.045*	
H23B	0.2997	0.5423	0.9929	0.045*	
N1	0.20188 (13)	0.70606 (14)	1.15357 (13)	0.0331 (3)	
O1	0.38397 (12)	0.65566 (13)	1.22367 (11)	0.0397 (2)	
O2	0.34760 (15)	0.81621 (16)	1.38221 (13)	0.0570 (4)	
O3	−0.19532 (13)	0.77004 (13)	0.75104 (14)	0.0476 (3)	
O4	0.04207 (15)	0.87474 (16)	0.84512 (19)	0.0707 (4)	
O5	−0.17187 (14)	0.40133 (14)	0.51266 (12)	0.0483 (3)	
O6	−0.17445 (18)	0.59307 (18)	0.49278 (15)	0.0668 (4)	
Br1	0.429587 (19)	0.76941 (2)	0.99976 (2)	0.05385 (8)	

### Atomic displacement parameters (Å^2^)

**Table:**

	*U*^11^	*U*^22^	*U*^33^	*U*^12^	*U*^13^	*U*^23^
C2	0.0370 (7)	0.0337 (8)	0.0387 (8)	0.0197 (6)	0.0174 (6)	0.0204 (7)
C3	0.0481 (9)	0.0488 (10)	0.0408 (9)	0.0256 (8)	0.0221 (8)	0.0219 (8)
C4	0.0559 (10)	0.0622 (12)	0.0581 (12)	0.0346 (10)	0.0359 (10)	0.0291 (10)
C5	0.0445 (9)	0.0635 (12)	0.0712 (13)	0.0335 (9)	0.0335 (9)	0.0392 (11)
C6	0.0344 (7)	0.0478 (10)	0.0531 (10)	0.0204 (7)	0.0174 (7)	0.0311 (8)
C7	0.0342 (7)	0.0320 (7)	0.0414 (8)	0.0164 (6)	0.0152 (6)	0.0224 (7)
C8	0.0346 (7)	0.0316 (7)	0.0334 (7)	0.0160 (6)	0.0114 (6)	0.0184 (6)
C9	0.0361 (7)	0.0323 (7)	0.0299 (7)	0.0182 (6)	0.0112 (6)	0.0163 (6)
C10	0.0384 (7)	0.0385 (8)	0.0335 (8)	0.0202 (7)	0.0123 (6)	0.0185 (7)
C11	0.0334 (7)	0.0500 (10)	0.0342 (8)	0.0223 (7)	0.0086 (6)	0.0221 (7)
C12	0.0541 (10)	0.0722 (14)	0.0528 (11)	0.0435 (10)	0.0189 (9)	0.0271 (10)
C13	0.0495 (10)	0.0813 (15)	0.0576 (12)	0.0335 (10)	0.0208 (9)	0.0497 (11)
C14	0.0430 (9)	0.0559 (12)	0.0664 (14)	0.0151 (9)	0.0151 (9)	0.0268 (11)
C15	0.0340 (7)	0.0369 (8)	0.0340 (8)	0.0155 (6)	0.0096 (6)	0.0181 (7)
C16	0.0311 (7)	0.0401 (8)	0.0356 (8)	0.0142 (6)	0.0084 (6)	0.0209 (7)
C17	0.0402 (8)	0.0410 (9)	0.0419 (9)	0.0148 (7)	0.0079 (7)	0.0256 (8)
C18	0.0744 (14)	0.0448 (11)	0.0804 (16)	0.0348 (11)	0.0287 (12)	0.0297 (11)
C19	0.0846 (18)	0.0762 (19)	0.129 (3)	0.0495 (16)	0.0475 (19)	0.0222 (18)
C20	0.0338 (7)	0.0545 (11)	0.0392 (9)	0.0184 (7)	0.0104 (7)	0.0268 (8)
C21	0.0551 (10)	0.0651 (13)	0.0308 (9)	0.0161 (10)	0.0135 (8)	0.0163 (9)
C22	0.129 (3)	0.0657 (16)	0.0488 (14)	0.0332 (17)	0.0320 (15)	0.0117 (12)
C23	0.0413 (7)	0.0407 (8)	0.0314 (8)	0.0238 (7)	0.0135 (6)	0.0164 (7)
N1	0.0359 (6)	0.0358 (7)	0.0297 (6)	0.0204 (5)	0.0115 (5)	0.0165 (5)
O1	0.0410 (5)	0.0488 (7)	0.0298 (5)	0.0282 (5)	0.0088 (5)	0.0179 (5)
O2	0.0569 (7)	0.0709 (9)	0.0311 (6)	0.0389 (7)	0.0105 (6)	0.0118 (6)
O3	0.0427 (6)	0.0366 (6)	0.0566 (8)	0.0189 (5)	0.0121 (6)	0.0220 (6)
O4	0.0441 (7)	0.0440 (8)	0.0922 (12)	0.0081 (6)	0.0071 (8)	0.0281 (8)
O5	0.0547 (7)	0.0486 (7)	0.0314 (6)	0.0190 (6)	0.0119 (5)	0.0178 (6)
O6	0.0823 (10)	0.0742 (10)	0.0452 (8)	0.0385 (9)	0.0118 (7)	0.0387 (8)
Br1	0.05092 (11)	0.05899 (14)	0.05578 (13)	0.02350 (9)	0.02807 (10)	0.02914 (10)

### Geometric parameters (Å, °)

**Table:**

C2—C3	1.389 (2)	C14—H14A	0.96
C2—C7	1.393 (2)	C14—H14B	0.96
C2—N1	1.4057 (17)	C14—H14C	0.96
C3—C4	1.390 (2)	C15—C16	1.331 (2)
C3—H3	0.93	C15—H15	0.93
C4—C5	1.383 (3)	C16—C20	1.489 (2)
C4—H4	0.93	C16—C17	1.493 (2)
C5—C6	1.374 (3)	C17—O4	1.189 (2)
C5—H5	0.93	C17—O3	1.321 (2)
C6—C7	1.399 (2)	C18—C19	1.452 (3)
C6—H6	0.93	C18—O3	1.454 (2)
C7—C8	1.439 (2)	C18—H18A	0.97
C8—C9	1.365 (2)	C18—H18B	0.97
C8—C15	1.458 (2)	C19—H19A	0.96
C9—N1	1.4017 (19)	C19—H19B	0.96
C9—C23	1.4701 (19)	C19—H19C	0.96
C10—O2	1.183 (2)	C20—O6	1.198 (2)
C10—O1	1.3199 (18)	C20—O5	1.318 (2)
C10—N1	1.409 (2)	C21—O5	1.450 (2)
C11—O1	1.4929 (17)	C21—C22	1.471 (4)
C11—C14	1.498 (3)	C21—H21A	0.97
C11—C12	1.504 (2)	C21—H21B	0.97
C11—C13	1.507 (2)	C22—H22A	0.96
C12—H12A	0.96	C22—H22B	0.96
C12—H12B	0.96	C22—H22C	0.96
C12—H12C	0.96	C23—Br1	1.9654 (17)
C13—H13A	0.96	C23—H23A	0.97
C13—H13B	0.96	C23—H23B	0.97
C13—H13C	0.96		
			
C3—C2—C7	122.51 (14)	H14B—C14—H14C	109.5
C3—C2—N1	129.77 (15)	C16—C15—C8	127.53 (15)
C7—C2—N1	107.63 (12)	C16—C15—H15	116.2
C2—C3—C4	116.62 (17)	C8—C15—H15	116.2
C2—C3—H3	121.7	C15—C16—C20	121.44 (15)
C4—C3—H3	121.7	C15—C16—C17	122.82 (15)
C5—C4—C3	121.64 (17)	C20—C16—C17	115.34 (14)
C5—C4—H4	119.2	O4—C17—O3	125.11 (17)
C3—C4—H4	119.2	O4—C17—C16	122.78 (16)
C6—C5—C4	121.34 (15)	O3—C17—C16	112.10 (14)
C6—C5—H5	119.3	C19—C18—O3	109.1 (2)
C4—C5—H5	119.3	C19—C18—H18A	109.9
C5—C6—C7	118.48 (17)	O3—C18—H18A	109.9
C5—C6—H6	120.8	C19—C18—H18B	109.9
C7—C6—H6	120.8	O3—C18—H18B	109.9
C2—C7—C6	119.38 (14)	H18A—C18—H18B	108.3
C2—C7—C8	107.57 (12)	C18—C19—H19A	109.5
C6—C7—C8	133.01 (15)	C18—C19—H19B	109.5
C9—C8—C7	107.60 (13)	H19A—C19—H19B	109.5
C9—C8—C15	124.17 (13)	C18—C19—H19C	109.5
C7—C8—C15	128.12 (13)	H19A—C19—H19C	109.5
C8—C9—N1	109.10 (12)	H19B—C19—H19C	109.5
C8—C9—C23	125.35 (14)	O6—C20—O5	125.09 (17)
N1—C9—C23	125.16 (13)	O6—C20—C16	122.46 (18)
O2—C10—O1	127.84 (15)	O5—C20—C16	112.44 (14)
O2—C10—N1	122.20 (14)	O5—C21—C22	107.12 (18)
O1—C10—N1	109.93 (13)	O5—C21—H21A	110.3
O1—C11—C14	110.16 (14)	C22—C21—H21A	110.3
O1—C11—C12	101.60 (13)	O5—C21—H21B	110.3
C14—C11—C12	111.45 (16)	C22—C21—H21B	110.3
O1—C11—C13	108.63 (13)	H21A—C21—H21B	108.5
C14—C11—C13	113.56 (17)	C21—C22—H22A	109.5
C12—C11—C13	110.76 (17)	C21—C22—H22B	109.5
C11—C12—H12A	109.5	H22A—C22—H22B	109.5
C11—C12—H12B	109.5	C21—C22—H22C	109.5
H12A—C12—H12B	109.5	H22A—C22—H22C	109.5
C11—C12—H12C	109.5	H22B—C22—H22C	109.5
H12A—C12—H12C	109.5	C9—C23—Br1	110.47 (11)
H12B—C12—H12C	109.5	C9—C23—H23A	109.6
C11—C13—H13A	109.5	Br1—C23—H23A	109.6
C11—C13—H13B	109.5	C9—C23—H23B	109.6
H13A—C13—H13B	109.5	Br1—C23—H23B	109.6
C11—C13—H13C	109.5	H23A—C23—H23B	108.1
H13A—C13—H13C	109.5	C9—N1—C2	108.08 (12)
H13B—C13—H13C	109.5	C9—N1—C10	129.37 (12)
C11—C14—H14A	109.5	C2—N1—C10	122.55 (13)
C11—C14—H14B	109.5	C10—O1—C11	120.94 (12)
H14A—C14—H14B	109.5	C17—O3—C18	116.27 (15)
C11—C14—H14C	109.5	C20—O5—C21	116.52 (15)
H14A—C14—H14C	109.5		
			
C7—C2—C3—C4	−1.5 (3)	C17—C16—C20—O6	9.1 (2)
N1—C2—C3—C4	−177.75 (17)	C15—C16—C20—O5	15.1 (2)
C2—C3—C4—C5	0.7 (3)	C17—C16—C20—O5	−171.97 (14)
C3—C4—C5—C6	0.8 (3)	C8—C9—C23—Br1	101.19 (16)
C4—C5—C6—C7	−1.6 (3)	N1—C9—C23—Br1	−70.93 (17)
C3—C2—C7—C6	0.8 (2)	C8—C9—N1—C2	0.55 (17)
N1—C2—C7—C6	177.74 (14)	C23—C9—N1—C2	173.76 (14)
C3—C2—C7—C8	−177.20 (15)	C8—C9—N1—C10	−179.59 (15)
N1—C2—C7—C8	−0.23 (17)	C23—C9—N1—C10	−6.4 (3)
C5—C6—C7—C2	0.8 (2)	C3—C2—N1—C9	176.49 (17)
C5—C6—C7—C8	178.15 (17)	C7—C2—N1—C9	−0.18 (17)
C2—C7—C8—C9	0.57 (17)	C3—C2—N1—C10	−3.4 (3)
C6—C7—C8—C9	−177.01 (17)	C7—C2—N1—C10	179.95 (14)
C2—C7—C8—C15	176.90 (15)	O2—C10—N1—C9	164.58 (17)
C6—C7—C8—C15	−0.7 (3)	O1—C10—N1—C9	−17.1 (2)
C7—C8—C9—N1	−0.68 (17)	O2—C10—N1—C2	−15.6 (3)
C15—C8—C9—N1	−177.20 (14)	O1—C10—N1—C2	162.71 (14)
C7—C8—C9—C23	−173.88 (15)	O2—C10—O1—C11	−5.6 (3)
C15—C8—C9—C23	9.6 (2)	N1—C10—O1—C11	176.24 (13)
C9—C8—C15—C16	−136.69 (17)	C14—C11—O1—C10	−57.2 (2)
C7—C8—C15—C16	47.5 (3)	C12—C11—O1—C10	−175.45 (16)
C8—C15—C16—C20	−179.46 (14)	C13—C11—O1—C10	67.7 (2)
C8—C15—C16—C17	8.1 (3)	O4—C17—O3—C18	1.3 (3)
C15—C16—C17—O4	57.9 (3)	C16—C17—O3—C18	−179.61 (16)
C20—C16—C17—O4	−115.0 (2)	C19—C18—O3—C17	−156.5 (2)
C15—C16—C17—O3	−121.20 (17)	O6—C20—O5—C21	−1.9 (3)
C20—C16—C17—O3	65.95 (19)	C16—C20—O5—C21	179.18 (14)
C15—C16—C20—O6	−163.84 (17)	C22—C21—O5—C20	−177.9 (2)

### Hydrogen-bond geometry (Å, °)

**Table:**

*D*—H···*A*	*D*—H	H···*A*	*D*···*A*	*D*—H···*A*
C18—H18A···O4^i^	0.97	2.56	3.392 (3)	144

Symmetry codes: (i) −*x*, −*y*+2, −*z*+2.
